# Severe Myocardial Bridging-Induced Total Occlusion of the Left Anterior Descending (LAD) Artery Mimicking Wellens’ Syndrome: A Case Report

**DOI:** 10.7759/cureus.73123

**Published:** 2024-11-06

**Authors:** Nagarathna Shenoy, Karthik A Naik, Padmakumar R, Mahidhar Jeedigunta

**Affiliations:** 1 Cardiology, Kasturba Medical College, Manipal, Manipal, IND; 2 Cardiovascular Technology, Manipal College of Health Professions, Manipal Academy of Higher Education, Manipal, IND

**Keywords:** acute coronary syndrome, coronary angiography, left anterior descending artery, myocardial bridging, wellens syndrome

## Abstract

This case report describes a 66-year-old male with a three-month history of exertional chest pain that progressed from New York Heart Association (NYHA) class 2 to class 3 within one week, raising clinical concerns despite an unclear trigger. ECG findings showed biphasic T waves in leads V2 and V3 and T-wave inversions in leads V4 to V6, resembling the pattern seen in Wellens’ syndrome, which typically suggests critical left anterior descending (LAD) artery stenosis. However, initial assessments showed no significant atherosclerosis - a notable finding given the usual association of Wellens’ syndrome with atherosclerotic disease and its rarity in congenital anomalies. Coronary angiography and cardiac CT imaging revealed a severe myocardial bridge in the mid to distal LAD artery, leading to complete occlusion with preserved blood flow through a diagonal branch. These imaging modalities were essential for confirming the diagnosis by clearly depicting the extent of myocardial bridging and collateral circulation. This case underscores the importance of considering congenital anomalies, such as myocardial bridging, in the differential diagnosis of acute coronary syndromes, even when atherosclerosis is absent. Early recognition of these abnormalities is vital to ensure appropriate intervention and to prevent misdiagnosis.

## Introduction

A myocardial bridge is a congenital anomaly characterized by a segment of a coronary artery, most commonly the left anterior descending (LAD) artery, coursing through myocardial tissue bands rather than resting on the epicardial surface. This phenomenon typically involves the mid to distal portion of the LAD artery. First identified in 1961 in a report noting angiographic narrowing during systole, this discovery spurred advancements in diagnostic techniques such as coronary angiography and intravascular ultrasound (IVUS), now critical for detecting coronary anomalies. While traditionally considered benign, myocardial bridges can lead to severe complications, including acute coronary syndrome (ACS), myocardial infarction, or even cardiac arrest. Symptoms may include chest discomfort or pain, and management involves medications such as beta-blockers or calcium channel blockers and, in some cases, surgical intervention [1].

Wellens’ syndrome is an electrocardiographic pattern indicative of critical stenosis in the proximal LAD artery, marked by biphasic or deeply inverted T waves in the precordial leads, signaling a high risk of anterior myocardial infarction. Patients often present with symptoms resembling ACS, such as chest pain or tightness that may subside with rest. Early recognition of ECG changes is crucial to prevent adverse outcomes and ensure timely intervention [2]. Typically associated with atherosclerotic coronary artery disease, this case presents an unusual instance of severe myocardial bridging producing Wellens’ syndrome-like ECG changes, challenging the conventional link to atherosclerosis.

This report describes a rare instance of severe myocardial bridging causing complete LAD artery occlusion with ECG features resembling Wellens’ syndrome. A similar case has been reported by Avram et al. [3]. This case underscores the importance of considering myocardial bridging in the differential diagnosis of ACS, particularly in patients without significant coronary atherosclerosis.

## Case presentation

**Figure 1:**
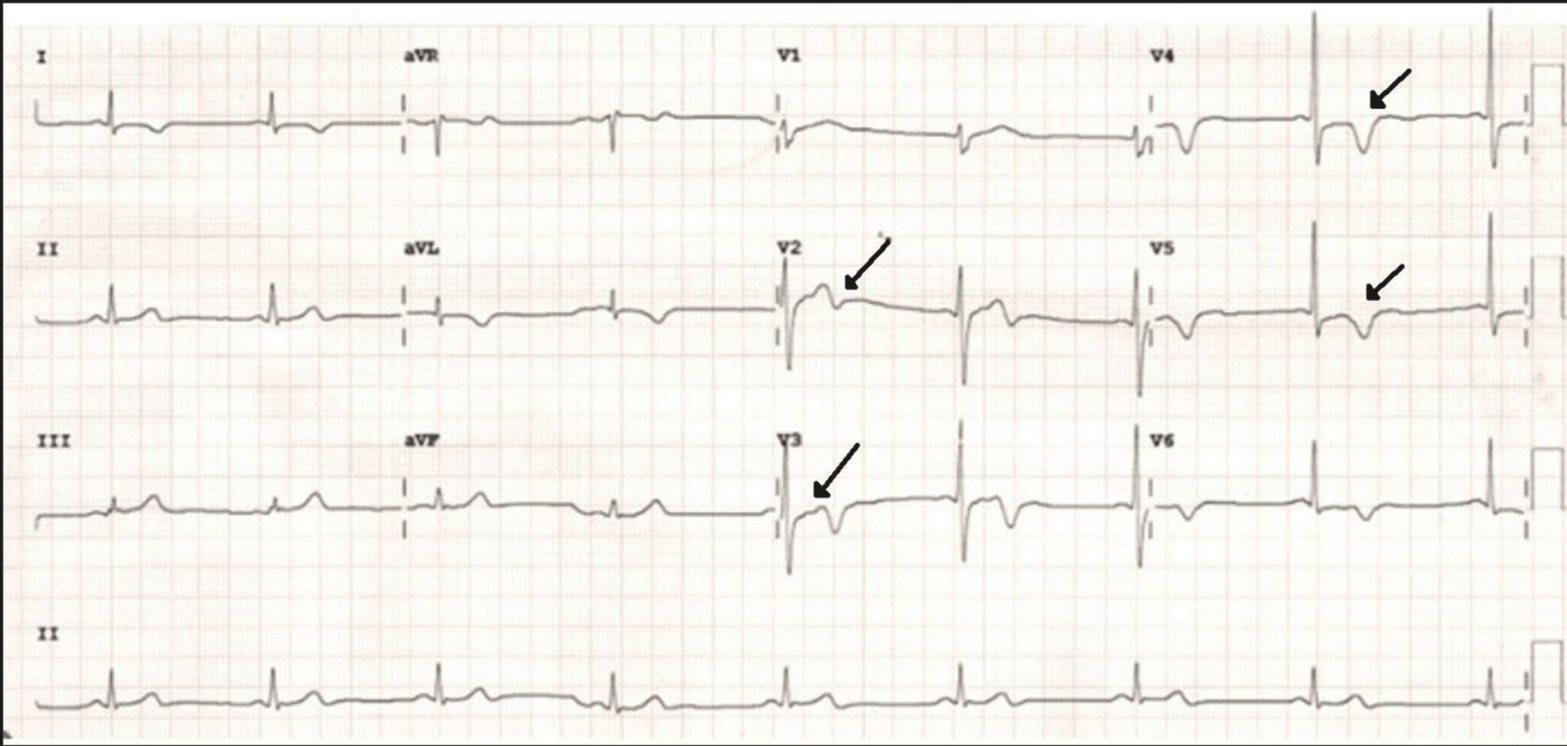
ECG demonstrating biphasic T waves in leads V2 and V3, indicative of Wellens sign

A 66-year-old male presented to the outpatient ward with a three-month history of exertional chest pain radiating to the left arm, which had progressed from New York Heart Association (NYHA) class 2 to class 3 over one week. He had no prior history of hypertension, diabetes, or coronary artery disease. Initial evaluation revealed a heart rate of 54 beats per minute, a blood pressure of 130/90 mmHg, and a respiratory rate of 19 breaths per minute. ECG showed sinus rhythm with biphasic T waves in leads V2 and V3 and T-wave inversions in leads V4 to V6, a pattern consistent with Wellens’ type A syndrome, suggestive of significant proximal LAD artery stenosis (Figure 1).

Laboratory investigations revealed a negative troponin T level of 0.007 ng/mL and an elevated serum creatinine level of 1.32 mg/dL. The lipid and liver panel tests were unremarkable (Table 1).

**Table 1:** Key laboratory findings in the patient

Test		Result	Normal range	Interpretation
Troponin T		0.007 ng/mL	Up to 0.020 ng/ml	Negative
Serum creatinine		1.30 mg/dL	0.7-1.2 mg/dL	Elevated (slightly abnormal)
Lipid panel	Total cholesterol (serum)	130 mg/dL	<200 mg/dL	Normal
	Total cholesterol/high-density lipoprotein cholesterol	4.2	Less than 5	
	Low-density lipoprotein cholesterol (serum)	79 mg/dL	>100 mg/dL	
	Tri glycerides (serum)	79 mg/dL	60-150 mg/dL	
Liver panel	Alanine transaminase (serum)	14.0 IU/L	Up to 41 IU/L	Normal
	Albumin (serum)	4.10 g/dL	3.5-5.2 g/dL	
	Alkaline phosphatase (serum)	54 U/L	40-130 U/L	
	Aspartate aminotransferase (serum)	22 IU/L	Up to 40 IU/L	
	Direct bilirubin (serum)	0.30 mg/dL	0.0-0.3 mg/dL	
	Total bilirubin (serum)	0.67 mg/dL	Up to 1.2 mg/dL	
	Globulin	3.50 g/dL	2.0-3.5 g/dL	
	Total protein (serum)	7.60 g/dL	6.4-8.3 g/dL	

**Figure 2:**
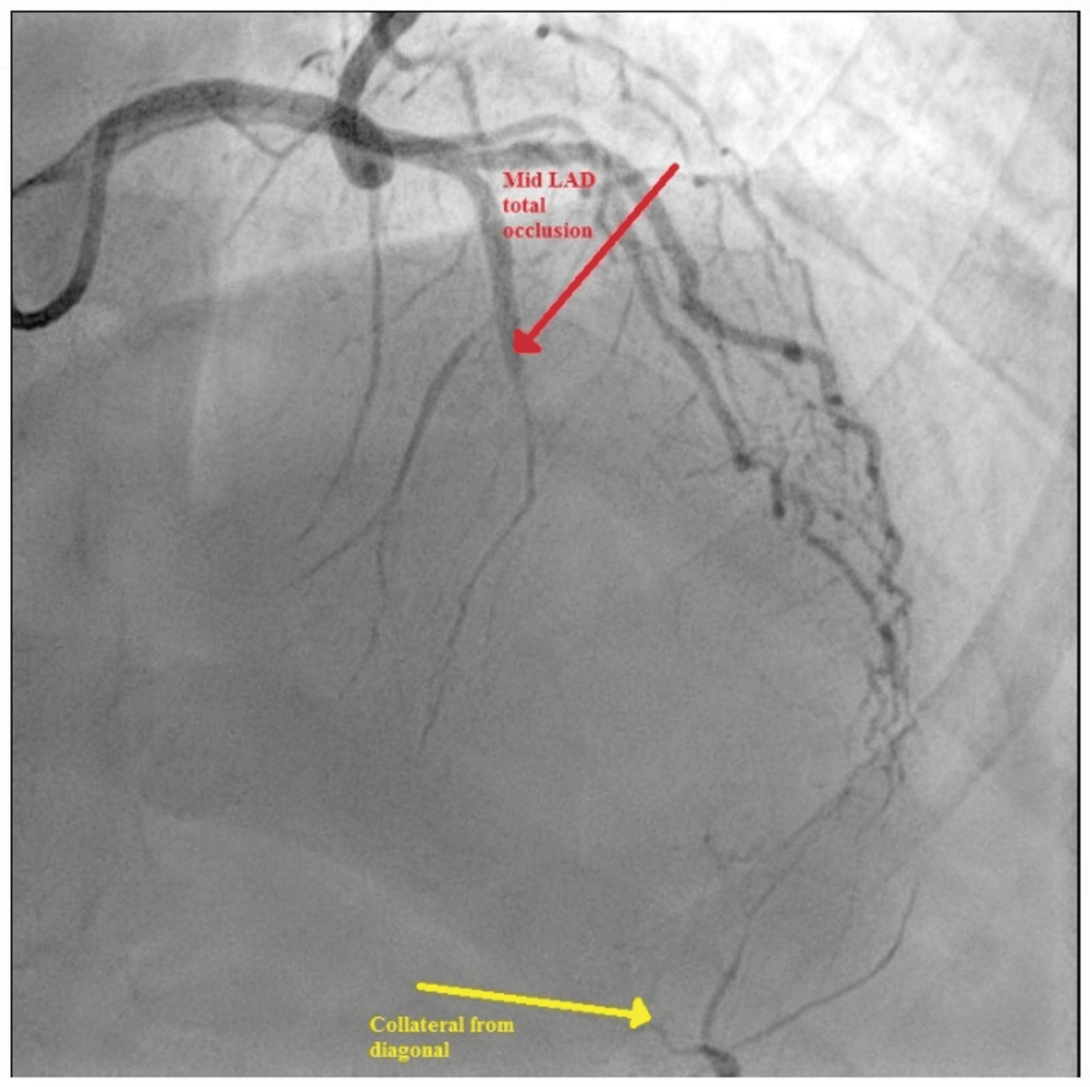
Coronary angiography showing long-segment myocardial bridging in the LAD artery with collateral flow to the distal LAD artery via the diagonal artery LAD, left anterior descending

Echocardiography (2D-TTE) revealed good biventricular function without abnormalities in regional wall motion. The patient was admitted to the cardiac care unit, and guideline-directed medical therapy was initiated for the tentative diagnosis of ACS. Coronary angiography was performed to assess coronary artery disease. The angiographic findings revealed a long segment of severe myocardial bridging in the mid to distal LAD artery, with no antegrade flow. The distal-most portion appeared to be supplied by collateral circulation arising from a major diagonal artery (Figure 2).

**Figure 3:**
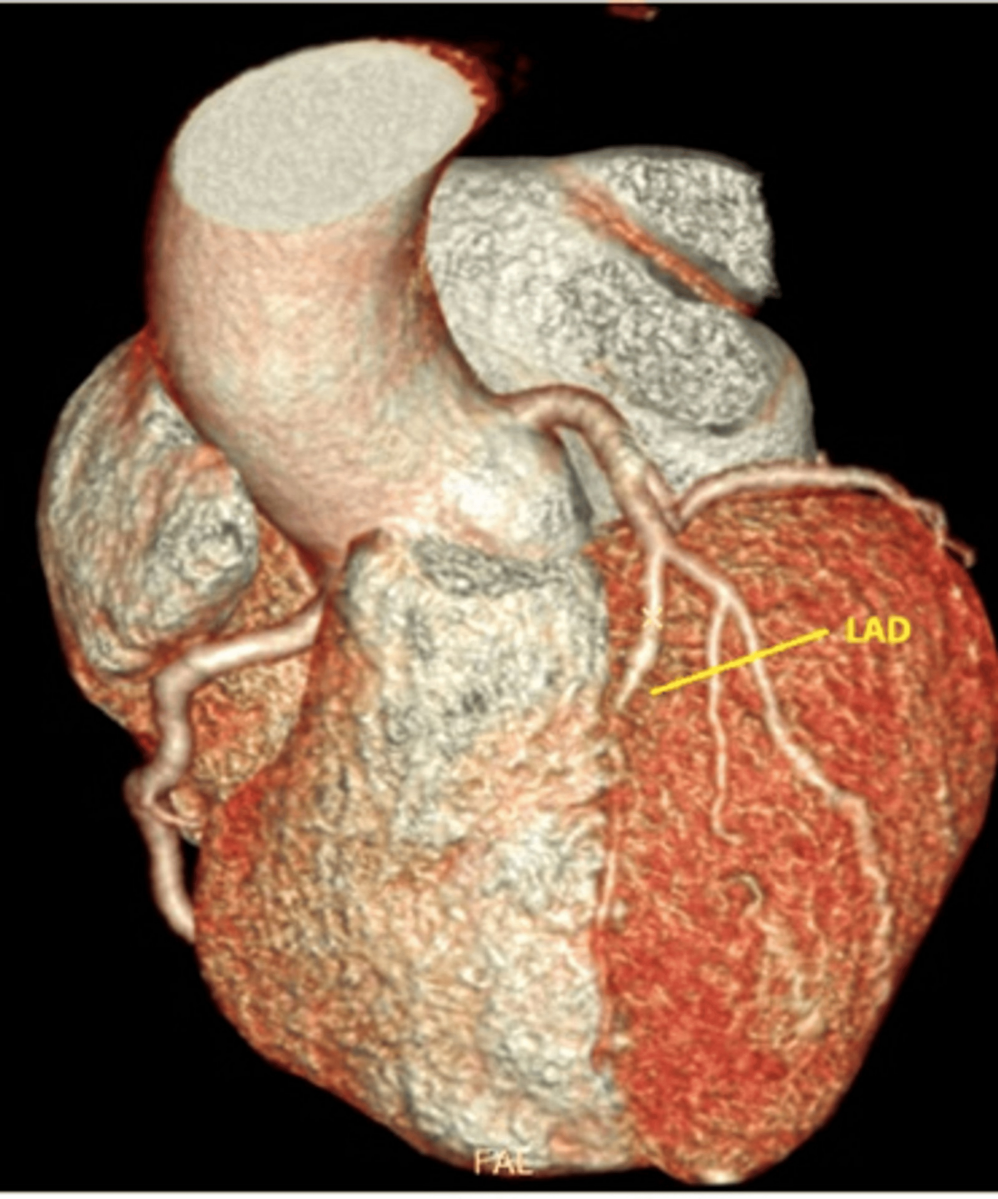
3D cardiac CT revealing a long-segment myocardial bridge in the LAD artery LAD, left anterior descending

Both the left circumflex artery and right coronary artery showed no significant lesions. To further delineate the anatomical details, a cardiac CT angiogram was performed, which confirmed the angiographic findings (Figure 3).

The patient was treated with a beta-blocker. During his three-month follow-up, he reported improvement and was able to carry out daily activities without discomfort.

## Discussion

Myocardial bridging is an anatomical phenomenon where a segment of a coronary artery, typically the LAD artery, tunnels through myocardial tissue. During ventricular systole, this tunneled segment is compressed, leading to transient narrowing or even complete occlusion, which reduces myocardial blood supply and can cause ischemia [4]. The reported incidence of myocardial bridging varies widely, from 1.5% to 16% in angiographic studies [5], while autopsy findings suggest a prevalence of 20-85%. This discrepancy arises because angiography may miss subtle or intermittent compression, whereas autopsies reveal all anatomical bridges, regardless of clinical significance [6]. Advanced imaging techniques such as IVUS and optical coherence tomography have improved detection, providing detailed visualization of coronary walls and luminal narrowing, often not visible in standard angiography [7].

The LAD artery is most commonly affected, although bridging can occur in other coronary arteries [8]. Structural compression, diastolic flow dynamics, and coronary vasoconstriction interplay in myocardial bridging, affecting coronary blood flow regulation and perfusion gradients, which increases ischemia risk [9]. Severe cases may lead to myocardial infarction, malignant arrhythmias, or sudden cardiac death [10]. IVUS and OCT provide detailed visualization of coronary walls and luminal narrowing, which may not be visible on standard angiography [7,11]. Additionally, cardiac MRI can also assess ischemia and myocardial viability, identifying regions with perfusion deficits and evaluating myocardial damage [12].

Wellens’ syndrome, first identified in 1982, is a characteristic ECG pattern linked to high-risk proximal LAD artery stenosis and, if untreated, can progress to extensive anterior myocardial infarction. It affects 14-18% of patients presenting with ACS [10,13]. Two T-wave patterns define it: type A, with deeply inverted T-waves in V2 and V3, and type B, with biphasic T-waves in these leads. These patterns typically point to a lesion between the first and second LAD artery septal branches [14]. Early diagnosis is essential for preventing adverse outcomes, and revascularization is recommended for a favorable prognosis [15].

In this case, we suggest that Wellens-like ECG changes were due to a long-segment myocardial bridge causing total LAD artery occlusion. While traditionally associated with atherosclerosis, this case challenges that paradigm by showing similar ECG findings resulting from myocardial bridging. Unlike previous reports of Wellens-like changes in myocardial bridging, this case involved complete LAD artery occlusion [3,16-19]. The patient’s presentation otherwise aligned with myocardial ischemia. The key distinction is the complete occlusion of the mid to distal LAD artery by myocardial bridging without atherosclerosis, underscoring the potential severity of this anomaly.

Pharmacologic therapy is the recommended first-line treatment for symptomatic myocardial bridging. Beta-blockers are frequently prescribed to reduce heart rate, myocardial contractility, and compression of the tunneled artery segment. This approach was effective in this patient, who reported significant improvement over a three-month follow-up, with no residual chest discomfort and a return to normal daily activities. Calcium channel blockers may be used for patients with bronchospasm risk, while antiplatelet therapy is reserved for those with atherosclerosis. Treatment aims to manage factors such as hypertension, left ventricular hypertrophy, tachycardia, and reduced diastolic filling. Vasodilators are typically avoided, as they may worsen perfusion gradients across the bridged segment. Multislice CT may also identify subclinical atherosclerosis prior to initiating antiplatelet therapy [20].

Notably, this case is remarkable for the absence of a history of comorbidities or evidence of coronary atherosclerosis. Instead, a severe long-segment bridge in the mid to distal LAD artery caused a total occlusion pattern, resulting in myocardial infarction-like symptoms. Treatment with a beta blocker led to marked improvement, and the patient reported being asymptomatic and able to perform daily activities comfortably at the three-month follow-up. This case highlights the value of advanced imaging modalities like coronary CT and IVUS for identifying myocardial bridging, emphasizing its potential for serious consequences, such as total occlusion and myocardial infarction, which necessitate tailored management.

This paper presents a unique case of myocardial bridging causing Wellens-like ECG changes and myocardial infarction symptoms due to complete LAD artery occlusion. It underscores the need to recognize myocardial bridging as a potential cause of ACS, even in the absence of obstructive atherosclerosis. Appropriate medical management and, if necessary, surgical intervention are essential for symptomatic patients with severe myocardial bridging.

## Conclusions

This unique case highlights the potential for severe myocardial bridging to induce complete arterial occlusion and mimic Wellens’ syndrome. This rare manifestation underscores the importance of considering myocardial bridging in the differential diagnosis of ACSs, even in the absence of obstructive coronary disease. Recognizing non-atherosclerotic causes of ACS can be diagnostically challenging, as standard diagnostic methods often fail to detect these structural anomalies. The mild elevation in creatinine observed in this case may reflect hemodynamic stress or early renal involvement, warranting careful monitoring. Additionally, the comprehensive interpretation of coronary angiography and cardiac CT findings was crucial in diagnosing myocardial bridging-induced LAD artery occlusion. Early recognition and a multidisciplinary, tailored approach, combining guideline-directed medical therapy and surgical evaluation, are essential for optimal management and preventing adverse outcomes in patients with hemodynamically significant myocardial bridging.
